# EEG Beta Power but Not Background Music Predicts the Recall Scores in a Foreign-Vocabulary Learning Task

**DOI:** 10.1371/journal.pone.0161387

**Published:** 2016-08-18

**Authors:** Mats B. Küssner, Annette M. B. de Groot, Winni F. Hofman, Marij A. Hillen

**Affiliations:** 1 Institut für Musikwissenschaft und Medienwissenschaft, Humboldt-Universität zu Berlin, Berlin, Germany; 2 Department of Psychology, University of Amsterdam, Amsterdam, The Netherlands; 3 Department of Medical Psychology, Academic Medical Center, University of Amsterdam, Amsterdam, The Netherlands; Universitat de Barcelona, SPAIN

## Abstract

As tantalizing as the idea that background music beneficially affects foreign vocabulary learning may seem, there is—partly due to a lack of theory-driven research—no consistent evidence to support this notion. We investigated inter-individual differences in the effects of background music on foreign vocabulary learning. Based on Eysenck’s theory of personality we predicted that individuals with a high level of cortical arousal should perform worse when learning with background music compared to silence, whereas individuals with a low level of cortical arousal should be unaffected by background music or benefit from it. Participants were tested in a paired-associate learning paradigm consisting of three immediate word recall tasks, as well as a delayed recall task one week later. Baseline cortical arousal assessed with spontaneous EEG measurement in silence prior to the learning rounds was used for the analyses. Results revealed no interaction between cortical arousal and the learning condition (background music vs. silence). Instead, we found an unexpected main effect of cortical arousal in the beta band on recall, indicating that individuals with high beta power learned more vocabulary than those with low beta power. To substantiate this finding we conducted an exact replication of the experiment. Whereas the main effect of cortical arousal was only present in a subsample of participants, a beneficial main effect of background music appeared. A combined analysis of both experiments suggests that beta power predicts the performance in the word recall task, but that there is no effect of background music on foreign vocabulary learning. In light of these findings, we discuss whether searching for effects of background music on foreign vocabulary learning, independent of factors such as inter-individual differences and task complexity, might be a red herring. Importantly, our findings emphasize the need for sufficiently powered research designs and exact replications of theory-driven experiments when investigating effects of background music and inter-individual variation on task performance.

## Introduction

### The effects of background music on foreign vocabulary learning

Background music—the sound accompanying many of our everyday activities, whether intentionally or not—has long been thought to influence our cognitive and affective behaviour in a variety of tasks in different environments, ranging from monotonous work in factories to studying foreign languages at home or in a classroom. Relevant to millions of people worldwide every day, foreign vocabulary learning is a cognitive task that has been shown to be sensitive to the learning environment. A critical review of the literature has suggested that background music, and particularly baroque and classical music, during learning gives rise to better memorization of vocabulary [1]. Some recent studies on vocabulary learning seem to confirm this observation. Kang and Williamson [2] reported that specifically composed music—simple tunes with drums, guitar and synthesizer sounds and little dynamic or rhythmic variation—improved learning Chinese vocabulary in a two-week, ecologically valid trial. A study by De Groot [3] suggested that soft baroque music during paired-associate vocabulary learning led to enhanced performance in a sub-sample of her participants. Another study by Ferreri and colleagues [4] has shown that memory performance is better when a list of words is learned with background music—an excerpt of guitar rock without lyrics—compared to environmental sounds or silence. However, some studies have failed to show any beneficial effect of background music on vocabulary learning [5–7]. The absence of this effect might to some extent be explained by inter-individual differences in personality [8, 9]. For example, De Groot [3] suggested that the lack of a general beneficial effect of background music on vocabulary learning in her study could be explained by participants’ differences in extraversion. Researchers have accumulated evidence in favour [8–15] and against [9, 10, 12, 14, 16, 17] this idea by investigating intro- and extraverts’ performance in various cognitive tasks. The most common theoretical framework used in these studies is Eysenck’s theory of personality [18], which forms the basis for the present study.

### Can the differential effects of background music on foreign vocabulary learning be explained by Eysenck’s theory of personality?

As discussed by Hagemann and colleagues [19], Eysenck suggested that differences in the personality trait extraversion are caused by differences in the basal level of cortical arousal (for another review see [20]). Eysenck hypothesized that extraverts, compared to introverts, possess a lower basal level of cortical arousal, or equivalently, a higher neurological threshold of cortical arousal. In other words, extraverts require more external stimulation than introverts to reach an optimal level of cortical arousal. At this optimal level, cognitive performance is supposed to be best, following the Yerkes-Dodson Law [21], which posits that cognitive performance is best at a medium level of cortical arousal and worst at low and high levels of arousal. However, the link between extraversion and cortical arousal remains speculative, as the literature provides evidence in favour [22, 23] and against [19, 24, 25] Eysenck’s theory. In the present study we examine whether this link exists by assessing both the personality trait extraversion and its suggested underlying cause, cortical arousal, which is usually measured as electroencephalogram (EEG) oscillatory power.

One recurring issue is the selection of the right range of frequencies in the EEG signal and their correlation with internal states of alertness and wakefulness. The classic assumption, dating back to the 1930s, is that alpha power (8–13 Hz) is inversely related to mental activity [25, 26]. According to this arousal model, increased alpha power reflects a more relaxed state or a decrease in cortical arousal. Ray and Cole [26] have shown that this model is too simple by providing evidence that alpha power is related to attentional demands, whereas beta power (14–35 Hz)—usually associated with states of wakefulness and activity—reflects emotional or cognitive processes. There is evidence that beta power is increased during cognitive tasks such as reading and subtraction [27] or sustained attention tests [28]. However, many personality researchers still use alpha power as a measure of cortical arousal, possibly because Eysenck himself (1994, p. 167, as cited in [20]) regarded EEG, and particularly alpha power, as the “standard measure of cortical arousal.” We therefore measured EEG oscillatory power in both alpha and beta bands.

### The present study

In two experiments, we tested the influence of inter-individual differences on foreign vocabulary learning during background music and silence. To that end, we recruited highly extravert and introvert participants and assessed their baseline cortical arousal by recording spontaneous EEG during silence and with their eyes open prior to a vocabulary-learning task.

The present study builds on de Groot’s [3] finding that background music, specifically Bach’s Brandenburg Concerto No. 4, enhances foreign vocabulary learning compared to silence. Crucially, the background music effect reported in her study generalized across the words to be learned but not across participants, hinting at the possibility that differences in participants’ extraversion score and/or basal cortical arousal—both of which were not assessed in her study—led to differential task performance. The design of our study is very similar to de Groot’s [3], using the same 64 word pairs and background music. In case extraversion and cortical arousal were not correlated in our data, we would proceed the analysis with cortical arousal instead of extraversion as a measure of inter-individual differences. Our hypotheses were thus as follows:

(1) extraverts show lower cortical arousal (i.e. more alpha and less beta power) than introverts during baseline measurement.
(2a) introverts’ performance in a word recall task is worse when learning occurred with background music as opposed to silence, whereas extraverts show similar or enhanced performance with background music compared to silence.
(2b) individuals with high cortical arousal (i.e. less alpha and more beta power) perform worse in a word recall task when background music was present during learning because we hypothesized the music to drive the cortical arousal beyond the optimal threshold. Similarly, we expected that individuals with low cortical arousal should be unaffected by background music or benefit from it because we hypothesized the music to drive the cortical arousal towards the optimal threshold.

Finally, we also explored the main effects of background music and cortical arousal on foreign vocabulary learning, and checked whether any effect, if present, still existed one week later.

## Experiment 1

### Methods

#### Participants

Thirty-three first-year psychology students at the University of Amsterdam participated, for which they were rewarded with course credits. One male participant was excluded from the analysis because of technical problems and data loss. All participants were admitted to the study based on their extreme scores (upper and lower three stanines) on the extraversion scale of the “Vijf Persoonlijkheidsfactoren test, 5PFT” [29, 30], assessed previously during an obligatory part of the curriculum and confirmed on the day of the experiment six months later. One female participant was excluded because her score had dropped from the extravert to the introvert range within these six months. Of the remaining 31 participants (mean age: 21.06 years, SD = 3.53 years, range: 19–38 years), fifteen (eleven females) had a high extraversion score (upper three stanines) with a mean score of 82.53 (SD = 6.06; range: 71–94 out of 100) and were labelled as “extravert”. Sixteen participants (seven females) scored within the lower three stanines of the extraversion scale (mean score: 46.25; SD = 7.57; range: 29–56 out of 100) and were labelled as “introvert”. The extraversion scores were significantly different between intro- and extraverts, *t*(29) = 14.674, *p* < .001, *d* = 5.450. All participants were native speakers of Dutch and each participant signed an informed consent form. The study was approved by the Ethics Committee of the Psychology Department of the University of Amsterdam.

#### Materials

The stimulus set (for details, see [3]) consisted of 64 pairs of letter strings, one letter string being a Dutch word, the other a non-word. The set was varied, containing concrete and abstract Dutch words, Dutch words of high and low frequency, and with the non-words in the stimuli consisting of letter strings that agreed with Dutch phonology and orthography as well as letter strings that violated Dutch phonology and orthography.

The background music consisted of the first two movements of J. S. Bach’s Brandenburg Concerto No. 4 in G major (BWV 1049), and was the same recording as in de Groot’s study [3]. The sound level varied between 36 and 53 dB(A), with a mean sound level of 46.8 dB(A) over the entire 10-minute fragment. In the silence condition, the mean sound level varied between 30 and 33 dB(A).

#### Apparatus and EEG recording

Baseline EEG was recorded with a Neuroscan amplifier (NeuroScan Inc., Hernon, USA), using a high pass filter of 0.03 Hz, a low pass filter of 100 Hz and a sampling frequency of 256 Hz. Six EEG derivations were recorded at P3, P4, C3, C4, O1 and O2, with A1 and A2 as linked reference. To control for vertical and horizontal eye movements EOG was recorded from two electrodes above and below one of the eyes (VEOG), and two electrodes on the outer canthi of both eyes (HEOG), respectively.

#### Procedure

After completing the ‘Vijf Persoonlijkheidsfactoren Test’(5PFT) by Elshout and Akkerman [29, 30] participants were attached to the EEG device. Each participant was exposed to two sessions of learning and testing, plus a retest one week later. In one of the sessions participants had to perform the vocabulary-learning task with background music (the fragment was played in a loop) and in the other session learning was performed in silence. The order of the silence and music conditions was randomized between participants, and the 64 stimulus pairs were evenly distributed over the music and the silence condition, 32 stimulus pairs in each condition. The order of presentation of the two subsets was randomized. Each session was preceded by a 10-minute recording of the EEG to measure baseline arousal. The learning session with music was preceded by an arousal measurement with music while the learning session in silence was preceded by an arousal measurement in silence. Participants were instructed to sit silently with their eyes open during the arousal measurements. For the analyses, only the arousal measurement in silence was used, as is common practice for baseline EEG measurements [19, 22]. The arousal measurement with background music prior to learning with background music was included in the experimental procedure to ensure that the two conditions (learning in silence and with background music) were framed as similar as possible. During the learning task itself (whether in silence or with background music) cortical arousal was not measured.

Next, participants performed the vocabulary-learning task. They completed three learning rounds, each one immediately followed by a test. During each learning round participants were presented with 32 stimulus pairs (a Dutch word paired with a non-word) on a screen. The Dutch word and the non-word appeared simultaneously next to each other. Stimulus pairs were presented in random order, for 10 seconds each. Each learning round was followed by a test that involved the presentation of the 32 Dutch words on the screen. Participants were instructed to verbally reproduce the corresponding non-word, and the experimenter typed in the participants’ responses. Only perfectly recalled words counted as correct, and all tests were taken in silence. This procedure of a 10-minute arousal measurement followed by three learning rounds, each followed by a test, was then repeated in the other condition (music or silence).

One week later a retest was run, during which all 64 Dutch words from both the music and silence condition were presented once more in two blocks, one block consisting of the 32 words previously presented in the music condition, the other containing the 32 words presented in silence before. The participants had to reproduce the corresponding non-words again. The presentation order of the two blocks was randomized between participants. This retest was not preceded by an additional round of learning and, just like all tests the week before, it was conducted without background music.

#### Analysis of the EEG data

The EEG data were edited manually to remove muscle artifacts and artifacts caused by environmental noise. The Gratton, Coles and Donchin algorithm [31] was used to perform eye movement correction. Absolute spectral power calculated from the edited EEG data was then analyzed, using Fast Fourier Transform analysis with a Hamming window on 4-second time windows with an overlap of 2 seconds. Absolute spectral power was calculated for each electrode location. For statistical analysis the percentage of the total power in the following frequency bands was used: alpha I (7.5–10 Hz), alpha II (10.5–12 Hz), beta I (14–25 Hz), and beta II (25–35 Hz). Participants were divided into low and high arousal groups for both alpha (I+II) and beta (I+II) power, based on a median split of the mean percentage of power over all electrode sites.

#### Statistical analysis

To test Hypothesis 1, a set of 24 one-way ANOVAs was run to compare intro- and extraverts’ mean percentage of power in the four alpha and beta bands over all six electrode sites. Given the result of this analysis (see Results below) we excluded Hypothesis 2a from the analysis and only tested Hypothesis 2b. To that end, two pairs of repeated-measures ANOVAs with the factors Condition (music vs. silence) and, respectively, Alpha Power (high vs. low) and Beta Power (high vs. low) were run on the recall scores of the tests that followed each learning round. Each pair consisted of one analysis by participants and one analysis by items. The dependent variable was the percentage of correct responses. To examine whether continued vocabulary learning increased recall scores as expected, we included a factor Test (Test 1, Test 2 and Test 3) as a manipulation check. In the analyses by participants, Condition and Test were within-subjects factors and Alpha/Beta Power was a between-subjects factor. In the analyses by items, Condition, Test and Alpha/Beta Power were all within-items factors. Unless stated otherwise, the analyses reported below concern the analyses by participants. If violated in any analysis, sphericity was Greenhouse-Geisser-corrected.

To keep the results sections concise, the analyses by items are only reported here if the results deviated from the corresponding analyses by participants (e.g., a non-significant result in the analysis by participants but a significant result in the analysis by items). Finally, *t*-tests were run to explore the main effects of Condition and Alpha/Beta Power on recall scores of the Retest.

### Results

#### Extraversion and cortical arousal

None of the 24 (frequency bands by electrode sites) ANOVAs on the power values revealed a significant difference at the Bonferroni-corrected alpha level (0.002083; see Table 1 for the mean percentage of power for introverts and extraverts per frequency band and electrode site). We therefore no longer considered Hypothesis 2a and proceeded by testing Hypothesis 2b.

**Table 1:** Experiment 1. Intro- and extraverts' mean percentage of power in alpha and beta bands over all six electrode sites.

		Introverts	Extraverts
		Mean (SD)	Mean (SD)
**P3**	**alpha I**	3.38 (2.92)	2.76 (3.72)
	**alpha II**	1.51 (1.47)	1.26 (1.22)
	**beta I**	3.28 (3.42)	2.60 (1.67)
	**beta II**	2.11 (2.20)	1.82 (1.67)
**P4**	**alpha I**	3.71 (2.60)	3.18 (4.70)
	**alpha II**	1.81 (1.65)	1.54 (1.53)
	**beta I**	3.31 (3.25)	2.49 (1.31)
	**beta II**	1.92 (2.07)	1.56 (1.19)
**C3**	**alpha I**	2.84 (2.30)	2.14 (2.54)
	**alpha II**	1.55 (1.71)	1.20 (1.25)
	**beta I**	2.97 (3.17)	2.33 (1.16)
	**beta II**	1.68 (1.97)	1.55 (1.05)
**C4**	**alpha I**	3.38 (2.19)	2.44 (2.46)
	**alpha II**	1.82 (1.70)	1.33 (1.09)
	**beta I**	3.24 (2.98)	2.60 (1.39)
	**beta II**	1.80 (1.86)	1.72 (1.49)
**O1**	**alpha I**	4.35 (8.22)	1.27 (1.03)
	**alpha II**	1.61 (2.35)	0.90 (1.02)
	**beta I**	3.67 (3.11)	2.28 (2.28)
	**beta II**	3.08 (2.64)	1.92 (2.63)
**O2**	**alpha I**	4.63 (5.53)	1.44 (0.77)
	**alpha II**	1.79 (1.44)	1.16 (0.95)
	**beta I**	4.04 (2.81)	2.55 (1.77)
	**beta II**	2.91 (2.54)	1.90 (1.81)

#### Manipulation check

The manipulation check revealed that vocabulary learning occurred as expected (*F*(1.262, 36.589) = 221.124, *p* < .001, partial η^2^ = .884). Participants’ mean scores increased from 13.11% (SEM = 1.54) in Test 1 to 36.71% (SEM = 3.30) in Test 2, reaching 56.41% (SEM = 3.86) in Test 3 (*p* < .001 for all pairwise comparisons).

#### Vocabulary learning and cortical arousal

There was no interaction between Condition and Alpha Power (*F*(1, 29) = 0.035, *p* = .852) and no main effect of Alpha Power (*F*(1, 29) = 0.004, *p* = .953).

There was no interaction between Condition and Beta Power (*F*(1, 29) = 0.007, *p* = .934), but a marginally significant main effect of Beta Power (*F*(1, 29) = 3.982, *p* = .055, partial η^2^ = .121), which was highly significant in the analysis by items (*F*(1, 31) = 64.011, *p* < .001, partial η^2^ = .674). Participants with high beta power (*M* = 40.53%, SEM = 3.68) performed better than participants with low beta power (*M* = 29.97%, SEM = 3.80). However, the effect disappeared in the retest, *t*(27.914) = 1.558, *p* = .131, *d* = 0.555. No difference was found between the music and the silence condition (*F*(1, 29) = 2.658, *p* = .114).

**Fig 1:**
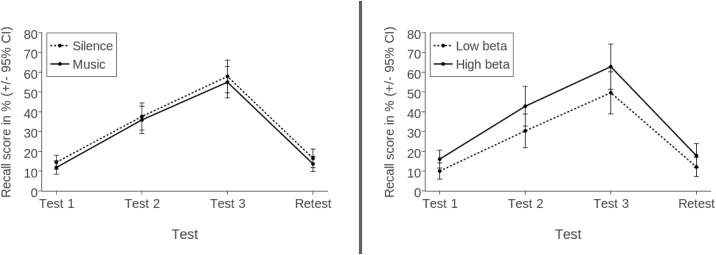
Mean recall scores (in %) of Experiment 1 by condition and beta power. Scores are based on analysis by participants.

An overview of participants’ mean recall scores, plotted separately for condition and beta power, can be seen in Fig 1.

### Discussion

There was no evidence for the hypothesized association between extraversion and cortical arousal, which fits with the mixed findings in the literature [19, 22–25]. Furthermore, the fact that one (excluded) participant’s extraversion score had dropped from the extravert to the introvert range within six months casts doubt on the reliability of our extraversion measure (which was based on questionnaire data, as extraversion measures usually are). To use a more objective measure of inter-individual differences, we excluded the ‘extraversion’ variable from the remainder of our analyses and focused on cortical arousal only.

The hypothesized interaction between cortical arousal and background music was not supported either. Instead, our results revealed an unexpected main effect of beta power. Participants with high beta power learned more vocabulary than those with low beta power in the short term. This effect just failed to reach significance in the analysis by participants but was highly significant in the analysis by items, suggesting that some inter-individual variance was unaccounted for (see Summary and General Discussion). Beta power has been related to cortical arousal [32, 33] and increased level of wakeful alertness [34]. Higher alertness might thus explain why participants with higher beta power outperform those with lower beta power. As the effect of beta power on cognitive task performance seems to be novel and is potentially very relevant, we conducted an exact replication of Experiment 1.

## Experiment 2

### Methods

Participant recruitment and compensation were identical to those in Experiment 1. Forty-two first-year psychology students at the University of Amsterdam participated in the study. None of them had participated in Experiment 1. One of them was excluded because she was a non-native speaker of Dutch, another because she was classified as “introvert” in the pretest and as “extravert” on the day of the experiment (see Experiment 1 for a similar classification reversal), and two participants (one male, one female) were excluded because of technical problems and data loss. Of the remaining 38 participants (mean age: 20.45 years, SD = 4.19 years, range: 18–43 years), eighteen (sixteen females) had a high extraversion score with a mean score of 73.89 (SD = 4.54; range: 65–81 out of 100) and were labelled as “extravert”. Twenty participants (nine females) scored low on the extraversion scale (mean score: 47.45; SD = 8.28; range: 33–61 out of 100) and were labelled as “introvert”. The extraversion scores were significantly different between intro- and extraverts, *t*(30.060) = 12.372, *p* < .001, *d* = 4.513. The hypotheses, materials, apparatus, EEG recording, experimental procedures and analyses on the data were all identical to those in Experiment 1, and the study was approved by the Ethics Committee of the Psychology Department of the University of Amsterdam.

### Results

#### Extraversion and cortical arousal

None of the 24 (frequency bands by electrode sites) ANOVAs on the power values revealed a significant difference at the Bonferroni-corrected alpha level (0.002083; see Table 2 for the mean percentage of power for introverts and extraverts per frequency band and electrode site). As in Experiment 1, we therefore no longer considered Hypothesis 2a and proceeded by testing Hypothesis 2b.

**Table 2:** Experiment 2. Intro- and extraverts' mean percentage of power in alpha and beta bands over all six electrode sites.

		Introverts	Extraverts
		Mean (SD)	Mean (SD)
**P3**	**alpha I**	2.56 (2.80)	1.86 (1.58)
	**alpha II**	1.10 (1.18)	1.19 (1.49)
	**beta I**	1.71 (1.46)	1.97 (1.95)
	**beta II**	0.82 (0.56)	1.55 (2.46)
**P4**	**alpha I**	2.98 (2.99)	3.03 (4.06)
	**alpha II**	1.09 (0.72)	1.41 (1.58)
	**beta I**	1.95 (1.37)	2.61 (2.49)
	**beta II**	0.96 (0.67)	1.70 (2.32)
**C3**	**alpha I**	2.00 (1.78)	2.58 (3.65)
	**alpha II**	0.85 (0.88)	1.19 (1.57)
	**beta I**	1.77 (1.46)	2.49 (2.45)
	**beta II**	0.97 (0.89)	1.72 (2.37)
**C4**	**alpha I**	2.79 (2.60)	2.64 (3.45)
	**alpha II**	1.25 (0.92)	1.18 (1.47)
	**beta I**	2.15 (1.73)	2.78 (2.79)
	**beta II**	0.96 (0.66)	1.83 (2.28)
**O1**	**alpha I**	3.38 (4.30)	2.78 (4.15)
	**alpha II**	1.32 (1.13)	1.22 (1.41)
	**beta I**	2.05 (1.57)	2.62 (2.83)
	**beta II**	1.16 (0.89)	1.84 (2.49)
**O2**	**alpha I**	3.54 (4.46)	2.43 (2.83)
	**alpha II**	1.59 (1.37)	1.35 (1.86)
	**beta I**	2.21 (1.92)	2.39 (3.07)
	**beta II**	1.08 (0.93)	1.37 (2.49)

#### Manipulation check

Vocabulary learning occurred as expected (*F*(1.309, 47.113) = 311.164, *p* < .001, partial η^2^ = .896). Participants’ mean scores increased from 13.49% (SEM = 1.43) in Test 1 to 37.05% (SEM = 2.85) in Test 2, reaching 55.02% (SEM = 3.28) in Test 3 (*p* < .001 for all pairwise comparisons).

#### Vocabulary learning and cortical arousal

There was no interaction between Condition and Alpha Power (*F*(1, 36) = 0.276, *p* = .602), and no main effect of Alpha Power (*F*(1, 36) = 0.397, *p* = .532) in the analysis by participants. However, there was a significant main effect of Alpha Power in the analysis by items (*F*(1, 31) = 8.603, *p* = .006, partial η^2^ = .217; high alpha power: *M* = 37.83%, SEM = 2.83; low alpha power: *M* = 34.90%, SEM = 2.97) which had not been found in Experiment 1, suggesting inter-individual differences within those with high alpha power: only a subset of them were better learners than those with low alpha power. However, this effect was not significant at the retest (*t*(36) = .442, *p* = .661, *d* = 0.143), indicating that this is not an enduring effect.

There was again no interaction between Condition and Beta Power (*F*(1, 36) = 0.742, *p* = .395). The main effect of Beta Power reported in Experiment 1 was non-significant in the analysis by participants (*F*(1, 36) = 0.412, *p* = .525), but significant in the analysis by items (*F*(1, 31) = 5.449, *p* = .026, partial η^2^ = .149; high beta power: *M* = 37.61%, SEM = 2.80; low beta power: *M* = 35.12%, SEM = 3.01), suggesting that a subset of participants with high beta power were better learners than those with low beta power. This effect did not last either, as shown by the analysis on the data of the retest, *t*(36) = .181, *p* = .857, *d* = 0.059.

**Fig 2:**
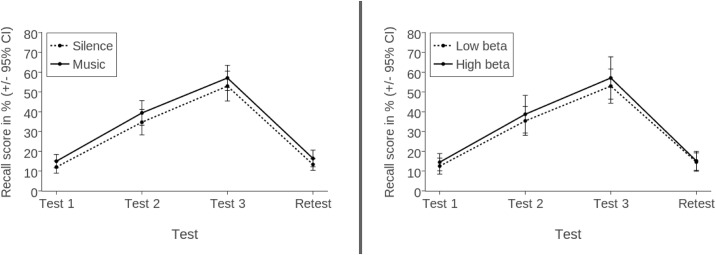
Mean recall scores (in %) of Experiment 2 by condition and beta power. Scores are based on analysis by participants.

Unlike in Experiment 1, however, there was a significant main effect of Condition (*F*(1, 36) = 4.610, *p* = .039, partial η^2^ = .114), indicating that participants performed better with background music (*M* = 37.14%, SEM = 2.56) compared to silence (*M* = 33.22%, SEM = 2.68). However, at retest this effect was no longer significant (silence: *M* = 13.32%, SEM = 1.46; background music: *M* = 16.37%, SEM = 2.07), *t*(37) = 1.755, *p* = .088, *d* = 0.276. Fig 2 displays an overview of participants’ mean recall scores, plotted separately for condition and beta power.

## Combined Analysis of Experiments 1 and 2

To further investigate the role of cortical arousal in foreign vocabulary learning, we merged the datasets of Experiment 1 and Experiment 2 for an analysis with greater statistical power (n = 69). Since combining these datasets would have required a reassignment of ten participants (4 for alpha power and 6 for beta power) based on a new median split, participants’ total alpha (I and II) and total beta (I and II) power were instead treated as predictors in a regression analysis, using the percentage of correct responses across background music and silence conditions as dependent variable.

**Fig 3:**
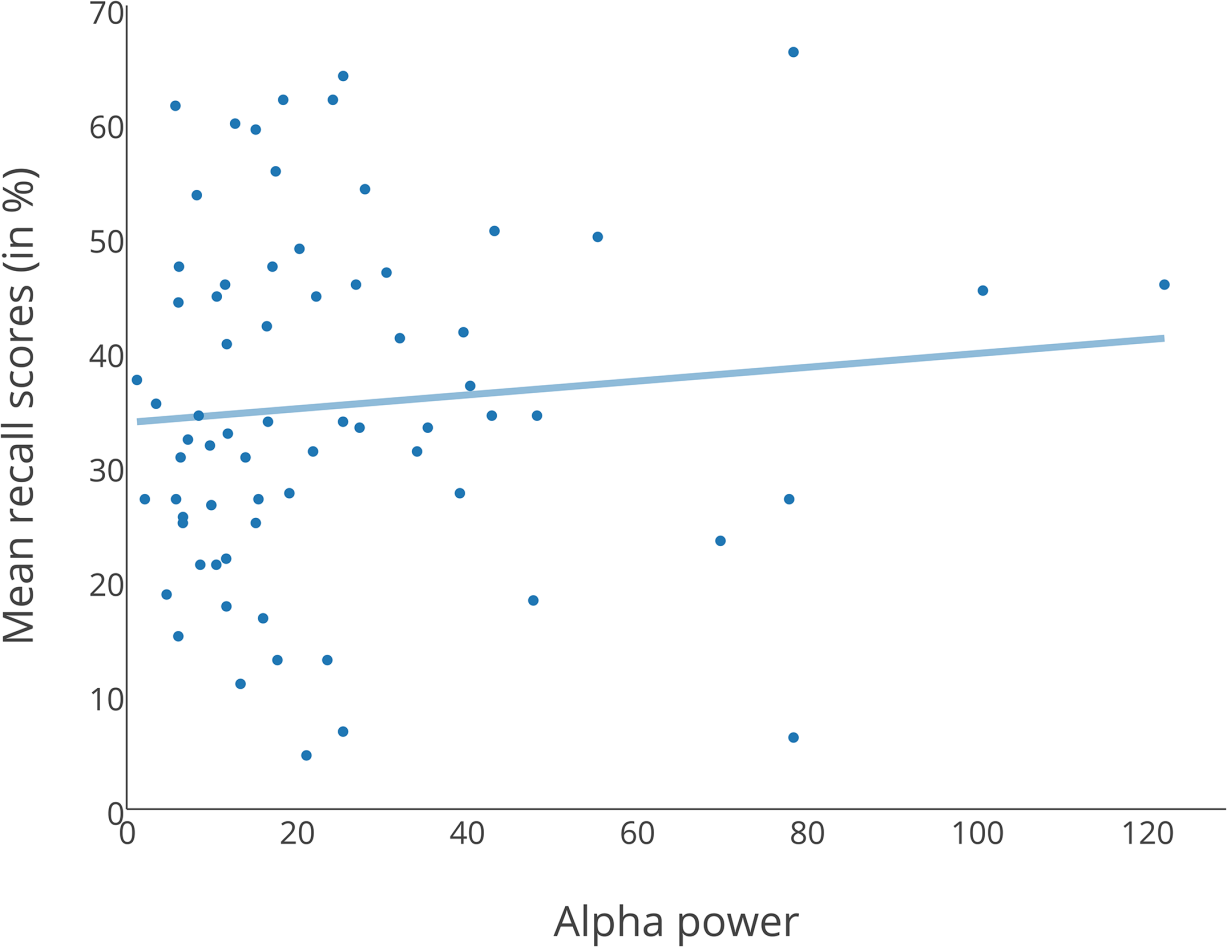
Regression plot with alpha power as predictor. Scores are based on analysis by participants.

**Fig 4:**
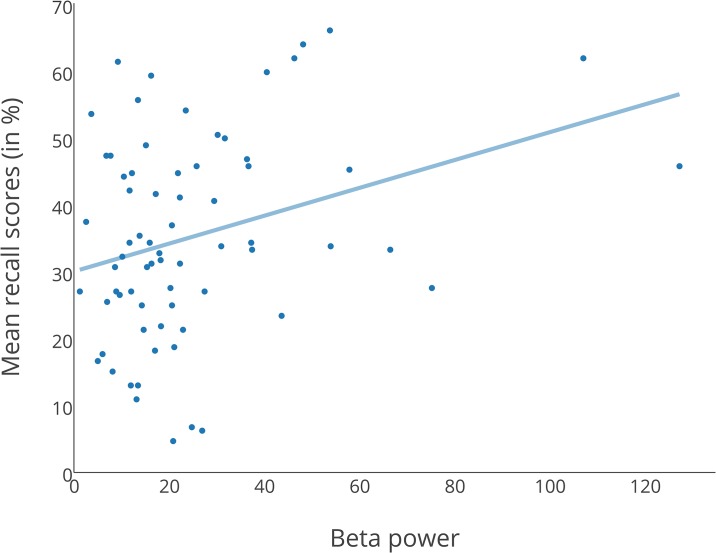
Regression plot with beta power as predictor. Scores are based on analysis by participants.

The overall fit of the regression model was R^2^ = .105, *F*(2, 66) = 3.853, *p* = .026. Beta power, but not alpha power, was a significant predictor for the number of correctly recalled words, as shown in Table 3 and Figs 3 and 4. A 2 (music vs. silence) x 3 (Test 1, Test 2 and Test 3) repeated measures ANOVA on the combined dataset showed no main effect of Condition (*F*(1, 68) = 0.741, *p* = .392).

**Table 3:** Regression coefficients of alpha and beta power.

	Word recall		
	B	SE B	β
**Constant**	30.820	2.783	
**Alpha power**	–.076	.090	–.119n.s.
**Beta power**	.254	.096	.376*

Notes: Word recall *r* = .323, R^2^ = .105. n.s. (not significant) *p* = .404.

* *p* = .010.

## Summary and General Discussion

We investigated whether background music interacts with extraversion and its hypothesized underlying cause, cortical arousal, in a foreign vocabulary learning task. Based on Eysenck’s theory of personality, we expected that extraverts show lower cortical arousal (i.e. more alpha and less beta power) than introverts during baseline measurement. Since no evidence for this association was found, we focused on the more objective measure of inter-individual differences, cortical arousal, in the remainder of our analyses. In accordance with Eysenck’s theory, we predicted that individuals with high cortical arousal should perform worse with background music, whereas individuals with low cortical arousal should be unaffected by background music or even improve their performance. No consistent evidence was found that an excerpt of baroque music, which had been found to increase performance in a very similar vocabulary learning task in a subgroup of participants [3], worsens paired-associate learning of foreign language vocabulary in individuals with high cortical arousal or enhances it in individuals with low cortical arousal.

Conducting an experiment and its exact replication, we found no main effect of background music in Experiment 1, while in Experiment 2 background music during vocabulary learning led to enhanced performance in tests of immediate, but not delayed, word recall. With both datasets combined, and thus greater statistical power, this effect vanished. Furthermore, we revealed a main effect of beta power (as measured prior to the learning task in silence) in Experiment 1, which was highly significant in the analysis by items and marginally significant in the analysis by participants. Individuals with high beta power performed better than those with low beta power during immediate recall, but not a week later. Although this effect was only present in a subsample of individuals with high beta power in Experiment 2, a regression analysis on the combined dataset suggests that beta power can explain a small (10%) but significant amount of the variance in the recall scores. Taken together, these findings (a) emphasize the importance of exact replications when investigating the (evidently capricious) effects of background music on cognitive task performance, (b) highlight the need for sufficiently powered research designs and (c) support the call for more theory-driven hypothesis testing in this strand of research [35].

### Effects of background music

Several reasons may explain why the background music used in this study showed no consistent effect (for further discussions of the effect of background music on task performance, see recent review chapters by Schellenberg and Weiss [36] and Hallam and MacDonald [37]). First, it is worth considering the type of background music used. As Felix [1] demonstrated in her review, specific types of background music such as baroque or classical music appear to be more beneficial than others. But the fact that our baroque excerpt did not lead to enhanced performance suggests, in line with Felix’s argument, that categorisation by genres is of little help because of the differences found within one epoch or genre. It is more important to describe exactly what excerpt was used to be able to establish links to structural (melody, harmony) and performance (timing, dynamics) features. Further studies show that background music with familiar language lyrics has a short-term but no long-term detrimental effect on (foreign) vocabulary learning [5, 7].

A second possibly relevant factor is the appreciation of the background music [38] and any other psychophysiological effects it may have on the individual. For instance, Cassidy and Macdonald [14] showed that while neither low-arousing music with positive affect nor silence has a detrimental effect on cognitive task performance, high-arousing, negative music does.

Third, the average sound level of our musical excerpt may have been too low, preventing the induction of arousal in the listener. That background music can be used as a source of external stimulation to increase arousal in participants has been shown in several studies (e.g. [39–41]). However, there is evidence that some individuals may benefit from background music’s calming, rather than its arousing, effects [42, 43]. Črnčec and colleagues [43] suggest that background music in the classroom has no beneficial effects on children’s cognitive performance, but that children with special needs may well benefit from its calming effects by reducing arousal and increasing focus.

Fourth, background music may only affect complex cognitive tasks (see [9] for a discussion). One might argue that the vocabulary-learning task with background music was not complex enough to drive the cortical arousal beyond the optimal threshold, and hence no differential effects of background music on task performance were observed.

Regardless of the above explanations, the crucial point is that a main effect of background music was present in Experiment 2 but not in Experiment 1. Thus, any study revealing effects of (specific types of) background music on task performance should be replicated to avoid drawing premature conclusions [44].

### Effects of inter-individual differences

It has been suggested that finding evidence in favour of Eysenck’s theory of personality depends on various factors–sometimes as subtle as the time of the day the data are gathered [20], which was not controlled in the present study. Nevertheless, our finding that individuals with high beta power—whether generally (Experiment 1) or only a subgroup of individuals (Experiment 2)—are better vocabulary-learners, if only in the short term, warrants further investigation into the effects of cortical arousal on performance in cognitive tasks, i.c. in vocabulary learning by means of paired-associate learning.

One candidate for inter-individual variation—and a possible reason why some effects were only present on the item level—is musical training. For instance, Patston and Tippett [45] showed that musically trained individuals perform worse in a language comprehension task with background music compared to silence, whereas untrained individuals do not differ in these two conditions. It is possible that, accidentally, more musically trained participants took part in Experiment 1 than in Experiment 2, which would explain why in the former a beneficial main effect of background music was absent. The amount of musical training, which was not assessed in the present study, may thus be a crucial factor in experiments involving both music and cognitive processing.

Finally, selecting more extreme cases of cortical arousal, instead of assigning the participants to the low- and high-arousal groups based on a median split, might increase the sensitivity of the design. Consequently, the hypothesized interaction between cortical arousal and background music might then show up in the recall scores. Comparable analyses that contrasted groups of extremely extravert and introvert individuals rather than extremes in cortical arousal have in one case revealed an interaction [9] but not in another [16]. In fact, a further analysis including the recall data of 20 low-beta participants and 21 high-beta participants showed the interaction between cortical arousal and background music not to be significant (*p* = .127).

## Conclusion

Given the findings from the present study and other studies [35], we raise the question of whether searching for an effect of background music on vocabulary learning might be a red herring. At the very least it must be concluded that the effects of background music are unstable, as demonstrated in our study where, in Experiment 1, an effect of background music on vocabulary learning was absent, whereas in its exact replication (Experiment 2) a beneficial effect of background music was obtained. Even if such general effects exist, they might not be independent from other factors such as inter-individual differences and task complexity.

Although our findings provide hardly any support for Eysenck’s theory of personality, our results are in accordance with previous findings highlighting the role of many (subtle) factors that may interact with one another. What is novel and warrants further research is the main effect of cortical arousal in the beta band. Even though this effect was only fleeting, future studies might investigate the underlying mechanisms, its link with attention and alertness, and possible ways of manipulating cortical arousal through external stimulation.

## Supporting Information

S1 File Dataset of Experiment 1 for analyses by participants. (XLS)

S2 File Dataset of Experiment 1 for analyses by items. (XLS)

S3 File Dataset of Experiment 2 for analyses by participants. (XLS)

S4 File Dataset of Experiment 2 for analyses by items. (XLS)
