# Correction: EEG Beta Power but Not Background Music Predicts the Recall Scores in a Foreign-Vocabulary Learning Task

**DOI:** 10.1371/journal.pone.0163759

**Published:** 2016-09-22

**Authors:** The PLOS ONE Staff

The initials DW appear incorrectly in the Author Contributions. The correct contributions are: Conceptualization: AMBG WFH MAH. Data curation: MBK MAH. Formal analysis: MBK MAH. Investigation: MBK MAH. Methodology: AMBG WFH MAH. Resources: MBK AMBG WFH MAH. Software: MAH. Validation: MBK AMBG WFH MAH. Visualization: MBK. Writing—original draft: MBK. Writing—review & editing: MBK AMBG WFH MAH. The publisher apologizes for the error.
